# Cellular localization of kinin B_1 _receptor in the spinal cord of streptozotocin-diabetic rats with a fluorescent [N^α^-Bodipy]-des-Arg^9^-bradykinin

**DOI:** 10.1186/1742-2094-6-11

**Published:** 2009-03-26

**Authors:** Sébastien Talbot, Patrick Théberge-Turmel, Dalinda Liazoghli, Jacques Sénécal, Pierrette Gaudreau, Réjean Couture

**Affiliations:** 1 Department of Physiology, Faculty of Medicine, Université de Montréal, C.P. 6128, Succursale Downtown, Montréal, Québec, H3C 3J7, Canada; 2 The Montreal Neurological Institute and Hospital, McGill University, 3801 University Street, Montreal, Québec, H3A 2B4, Canada; 3 Laboratory of Neuroendocrinology of Aging, Centre Hospitalier de l'Université de Montréal Research Center, Angus Technopole, Montréal, Québec, H1W 4A4, Canada

## Abstract

**Background:**

The kinin B_1 _receptor (B_1_R) is upregulated by pro-inflammatory cytokines, bacterial endotoxins and hyperglycaemia-induced oxidative stress. In animal models of diabetes, it contributes to pain polyneuropathy. This study aims at defining the cellular localization of B_1_R in thoracic spinal cord of type 1 diabetic rats by confocal microscopy with the use of a fluorescent agonist, [Nα-Bodipy]-des-Arg^9^-BK (BdABK) and selective antibodies.

**Methods:**

Diabetes was induced by streptozotocin (STZ; 65 mg/kg, i.p.). Four days post-STZ treatment, B_1_R expression was confirmed by quantitative real-time PCR and autoradiography. The B_1_R selectivity of BdABK was determined by assessing its ability to displace B_1_R [^125^I]-HPP-desArg^10^-Hoe140 and B_2_R [^125^I]-HPP-Hoe 140 radioligands. The *in vivo *activity of BdABK was also evaluated on thermal hyperalgesia.

**Results:**

B_1_R was increased by 18-fold (mRNA) and 2.7-fold (binding sites) in the thoracic spinal cord of STZ-treated rats when compared to control. BdABK failed to displace the B_2_R radioligand but displaced the B_1_R radioligand (IC_50 _= 5.3 nM). In comparison, IC_50 _values of B_1_R selective antagonist R-715 and B_1_R agonist des-Arg^9^-BK were 4.3 nM and 19 nM, respectively. Intraperitoneal BdABK and des-Arg^9^-BK elicited dose-dependent thermal hyperalgesia in STZ-treated rats but not in control rats. The B_1_R fluorescent agonist was co-localized with immunomarkers of microglia, astrocytes and sensory C fibers in the spinal cord of STZ-treated rats.

**Conclusion:**

The induction and up-regulation of B_1_R in glial and sensory cells of the spinal cord in STZ-diabetic rats reinforce the idea that kinin B_1_R is an important target for drug development in pain processes.

## Background

Kinins are vasoactive peptides and central mediators acting through the activation of two G-protein-coupled receptors (R) denoted as B_1 _and B_2 _[1,2]. The B_2_R is widely and constitutively expressed in central and peripheral tissues and is activated by its preferential agonists bradykinin (BK) and Lys-BK. The B_1_R is activated by the active metabolites des-Arg^9^-BK and Lys-des-Arg^9^-BK and has a low level of expression in healthy tissues. The latter receptor is upregulated after exposure to pro-inflammatory cytokines, bacterial endotoxins, and hyperglycaemia-induced oxidative stress [3-7].

An important role for kinin B_1_R has been postulated in nociception and pain [8-10]. B_1_R knock out mice are less sensitive to pro-inflammatory pain stimuli and to spinal sensitization [11-13]. B_1_R partakes to mechanical and/or thermal hyperalgesia induced by cytokines [14,15] through peripheral protein kinase C activation [16] and in the formalin test [17,18]. It also contributes to neuropathic pain after peripheral nerve injury [18-23] or after the induction of type 1 diabetes with streptozotocin (STZ) [24-27] and type 2 diabetes with high glucose feeding [7,28,29]. Thermal hyperalgesia was evoked by intraspinal stimulation of B_1_R in STZ-diabetic rats [9].

Basal expression of B_1_R was reported in the rat and human spinal cord dorsal horn as well as in rat dorsal root ganglion and small caliber primary sensory neurons [30-32]. Autoradiographic B_1_R binding sites are increased and distributed all over the grey matter of the spinal cord after peripheral nerve injury [22] and in models of diabetes [7,29,33]. This spatial distribution of B_1_R binding sites suggests that this receptor is not limited to primary sensory afferents but could also be present on spinal cord microglia and astrocytes.

To consolidate the role of B_1_R in pain polyneuropathy, its cellular distribution was investigated in the spinal cord of STZ-induced B_1_R with a newly developed fluorescent agonist named [Nα-Bodipy]-des-Arg^9^-BK (BdABK). The B_1_R selectivity of BdABK was determined by assessing its ability to displace B_1_R ([^125^I]-HPP-desArg^10^-Hoe 140) and B_2_R ([^125^I]-HPP-Hoe 140) radioligands by autoradiography. Moreover, the displacement of BdABK fluorescent labeling by B_1_R antagonists (R-715 and SSR240612) was assessed by confocal microscopy. We also investigated the *in vivo *activity of BdABK in comparison with its native agonist on thermal hyperalgesia in both STZ-treated and control rats. Appropriate selective antibodies were used in confocal microscopy to co-localize B_1_R on astrocytes, microglia and sensory C fibers in STZ-diabetic rats. The induction and overexpression of B_1_R in the spinal cord of STZ-diabetic rats was confirmed by qPCR and autoradiography. Experiments were achieved 4 days after STZ administration because previous studies showed that spinal cord B_1_R was maximally up-regulated and engaged in thermal hyperalgesia 2 days after STZ treatment [9,33].

## Methods

### Animals and treatments

All research procedures and the care of the animals were in compliance with the guiding principles for animal experimentation as enunciated by the Canadian Council on Animal Care and were approved by the Animal Care Committee of our University. Male Sprague-Dawley rats (200–225 g, Charles River, St-Constant, Que., Canada) were housed two per cage, under controlled conditions of temperature (23°C) and humidity (50%), on a 12 h light-dark cycle and allowed free access to normal chow diet (Charles River Rodent) and tap water.

#### STZ treatment

Rats were used 5 days after their arrival and injected under low light with freshly prepared STZ (65 mg/kg; i.p.; Sigma-Aldrich, Oakville, ON, Canada). Age-matched controls were injected with vehicle (sterile saline 0.9%, pH. 7.0) [33]. Glucose concentrations were measured, with a commercial blood glucose-monitoring kit (Accusoft; Roche Diagnostics, Laval, Que., Canada), in blood samples obtained from the tail vein, in non-fasting animals, before STZ injection, and 4 days after treatment. Only STZ-treated rats whose blood glucose concentration was higher than 20 mM were considered as diabetic.

#### Synthesis of [N^α^-Bodipy]-des-Arg^9^-BK

BdABK was synthesized using 4,4-difluoro-5,7-dimethyl-4-bora-3a, 4a-diaza-*s*-indacene-3-propionic acid succinimidyl ester (BODIPY^® ^FL SE, Molecular Probes/Invitrogen Canada Inc, Burlington, ON; emission 510 nm) and des-Arg^9^-BK (Bachem Bioscience inc., King of Prussia, PA, USA). Des-Arg^9^-BK was solubilized in 100 mM NaHCO_3 _0.1 M (pH 8.4), at a concentration of 1 mg/ml and two equivalents of BODIPY^® ^FL SE, solubilized in degassed dimethyl sulfoxide, at a concentration of 5 mg/ml was added. Completion of the reaction was achieved in 2 h, at ambient temperature, under continuous agitation. The fluorescent peptide was lyophilized and purified by C18 reverse-phase HPLC as previously described [34,35]. The purity of the peptide was ≥ 98% as assessed by analytical HPLC (UV and fluorescence detection).

#### Tissue preparation for autoradiography and microscopy

Four days after injection of STZ, rats were anaesthetized with CO_2 _inhalation and then decapitated. Upper thoracic spinal cord (T3-T7) was removed and frozen in 2-methylbutane (cooled at -40°C following exposure to liquid nitrogen) and stored at -80°C. Few days later, spinal cords were mounted in a gelatin block and serially cut into 20-μm thick coronal sections with a cryostat. Thus the sections were thaw-mounted on 0.2% gelatin-0.033% chromium potassium sulfate-coated slides and kept at -80°C for 1 month to allow the adhesion of sections to the coverslip glasses.

### Confocal microscopy

#### Slides preparation

On the day of experiments, sections were thawed at room temperature for 10 min to enhance sections adhesion. They were pre-incubated for 10 min in 25 mM PIPES-NH_4_OH buffer (pH 7.4) to allow degradation of endogenous kinins which could occupy receptors. Sections were exposed for 90 min to 50 μM BdABK. Thereafter, slides were washed twice (1 min) in PIPES and fixed with 4% para-formaldehyde [36]. Slides were washed three times (5 min) and then exposed to 1 M of glycine for 90 min to eliminate autofluorescence from aldehyde-fixed tissue. Tissues were permeabilized for 45 min with 0.1% Triton X-100.

#### Immunolabeling protocol

Slides were incubated with a blocking buffer (25 mM PIPES buffer supplemented with 3% bovine serum albumin (BSA) and 3% donkey serum) to prevent non-specific labeling. Antibodies were diluted in blocking buffer. A direct marker of DNA (TOPRO-3; Molecular Probes, Eugene, OR) was used at concentration of 1:5000. Rabbit anti-Ionized calcium binding adapter molecule 1 (anti-IBA-1, Wako, Richmond, VA) at a concentration of 2 μg/ml was used to label microglia [37-39]. Chicken anti-Glial fibrillary acidic protein (anti-GFAP, Chemicon, Hornby, ON) at a concentration of 1:500 was used as a specific marker of astrocytes [40]. Rabbit anti-calcitonin-gene-related peptide (CGRP) (Chemicon, Hornby, ON) at a concentration of 1:2000 was used as marker of sensory C fibers [41]. Mouse anti-transient receptor potential vanilloid 1 (TRPV1) (Chemicon, Hornby, ON) at a concentration of 1 μg/ml was used to label capsaicin receptor expressed on primary afferents [42]. Secondary antibodies were rhodamine anti-mouse (Chemicon, Hornby, ON) 1:500; cy5 anti-chicken (Chemicon, Hornby, ON) 1:500 and rhodamine anti-rabbit (Chemicon, Hornby, ON) 1:500.

#### Coverslip and microscopy

Slides were washed 3 times (5 min), mounted with coverslip, fixed with mowiol (12 h at room temperature) and stored at -4°C for 1 month or used in confocal microscopy.

#### SYBR green-based quantitative RT-PCR

Four days after injection of STZ, rats were anaesthetized with CO_2 _inhalation and then decapitated. The thoracic spinal cord (T1-T2) was isolated and approximately 10 mg of tissue were put in RNA *later *stabilization reagent (QIAGEN, Valencia, CA, USA). Total RNA was extracted from tissue according to the manufacturer's instructions. First-strand cDNA synthesized from 400 ng total RNA with random hexamer primers was used as template for each reaction with the QuantiTect Rev Transcription Kit (QIAGEN). SYBR Green-based real-time quantitative PCR using Mx3000p device for signal detection (Stratagene, La Jolla, CA, USA) was performed as described [43]. PCR was performed in SYBR Green Master mix (QIAGEN) with 300 nM of each primer. For standardization and quantification, rat 18S was amplified simultaneously. The primer pairs were designed by Vector NTI software and used [6] (Table 1).

**Table 1:** PCR primer pairs used in this study

	Sequences	Position	Gen Bank
18S Forward	5' TCA ACT TTC GAT GGT AGT CGC CGT 3'	363 – 386	X01117
18S Reverse	5' TCC TTG GAT GTG GTA GCC GTT TCT 3'	470 - 447	

B_1 _receptor Forward	5' GCA GCG CTT AAC CAT AGC GGA AAT 3'	367 – 391	NM_030851
B_1 _receptor Reverse	5' CCA GTT GAA ACG GTT CCC GAT GTT 3'	478 - 454	

PCR conditions were as follows: 95°C for 15 min, followed by 46 cycles at 94°C for 15 s, 60°C for 30 s and 72°C for 30 s. The cycle threshold (Ct) value represents the cycle number at which a fluorescent signal rises statistically above background [44]. The relative quantification of gene expression was analyzed by the 2^-ΔΔCt ^method [45].

### Quantitative autoradiography

#### Specific binding sites of [^125^I]-HPP-desArg^10^-Hoe 140 and [^125^I]-HPP-Hoe 140

The radioligands for kinin B_1_R, HPP-desArg^10^-Hoe140 (3-(4 hydroxyphenyl) propionyl-desArg^9^-D-Arg^0 ^[Hyp^3^, Thi^5^, D-Tic^7^, Oic^8^]Bradykinin) and kinin B_2_R, HPP-Hoe140 (3-(4 hydroxyphenyl) propionyl-D-Arg^0 ^[Hyp^3^, Thi^5^, D-Tic^7^, Oic^8^]Bradykinin) were synthesized and kindly provided by Dr Witold Neugebauer (Dept Pharmacology, University of Sherbrooke, Sherbrooke, Que., Canada). They were iodinated by the chloramine T method [46]. On the day of experiments, sections were incubated at room temperature for 90 min in 25 mM PIPES-NH_4_OH buffer (pH 7.4) containing: 1 mM 1,10-phenanthroline, 1 mM dithiothreitol, 0.014% bacitracin, 0.1 mM captopril, 0.2% bovine serum albumin (protease free) and 7.5 mM magnesium chloride in the presence of 200 pM of [^125^I]-HPP-desArg^10^-Hoe 140 or [^125^I]-HPP-Hoe 140 (specific activity: 2000 cpm/fmol or 1212 Ci/mmol) [29,33]. Non-specific binding was determined in the presence of 1 μM of unlabeled B_1_R antagonist: R-715 (AcLys [D-βNal^7^, Ile^8^]des-Arg^9^-BK) [1] or of 1 μM of unlabeled B_2_R antagonist: Hoe 140 (Icatibant or JE 049, Jerini AG, Berlin, Germany) [47]. At the end of the incubation period, slides were transferred sequentially through four rinses of 4 min each in 25 mM PIPES (pH 7.4; 4°C) dipped for 15s in distilled water (4°C) to remove the excess of salts, and then air-dried. Kodak Scientific Imaging Films BIOMAX™ MR^® ^(Amersham Pharmacia Biotech Canada) were juxtaposed onto the slides in the presence of [^125^I]-microscales and exposed at room temperature for 7 days. The films were developed (GBX developer) and fixed (GBX fixer). Autoradiograms were quantified by densitometry using an MCID™ image analysis system (Imaging Research, St. Catharines, ON, Canada). A standard curve from [^125^I]-microscales was used to convert density levels into fentomoles per milligram of protein [48]. Specific binding was determined by subtracting values of nonspecific binding from that of total binding.

#### Specificity of BdABK

To assess the specificity of BdABK for B_1_R, competition curves were performed in autoradiography by incubating 200 pM of [^125^I]-HPP-desArg^10^-Hoe 140 with increasing concentrations (10^-10 ^to 10^-6 ^M) of R-715 (selective B_1_R antagonist, kindly provided by Dr Domenico Regoli, Pharmacology, University of Ferrara, Italy), des-Arg^9^-BK (dABK, selective B_1_R agonist, Bachem Bioscience inc., King of Prussia, PA, USA) and BdABK. Moreover, competition curves were performed by incubating 200 pM of [^125^I]-HPP-Hoe 140 with increasing concentrations (10^-10 ^to 10^-6 ^M) of Hoe 140 (selective B_2_R antagonist) and BdABK. Each concentration of each competitor was tested on 4 sections per rat from 7 different rats. Those sections were exposed to the film, and total binding was calculated as described above. Moreover, the specificity of BdABK was determined in confocal microscopy by the displacement of fluorescent labeling with the addition of 10^-5 ^M R-715 or SSR240612 [(2R)-2-[((3R)-3-(1,3-benzodioxol-5-yl)-3-[[(6-methoxy-2-naphthyl)sulfonyl]amino]propanoyl)amino]-3-(4- [2R, 6S)-2,6 dimethylpiperidinyl]methyl]phenyl)-N-isopropyl-N-ethylpropanamide hydrochloride] (kindly provided by Dr Pierre Carayon, Sanofi-Aventis, Montpellier, France) [18] to the incubation medium.

### Microglial cell culture

#### Primary cell culture method

Mixed glial cultures were prepared following the protocol of McCarthy and de Vellis [49] with some modifications. Briefly, forebrains were dissected out from one litter of 2-day-old Sprague-Dawley rat pups and the meninges were stripped off before enzymatic and mechanical dissociation. For enzymatic dissociation, HBSS containing 0.25% trypsin (Gibco 15090-046) was used. The tissue-trypsin suspension was incubated for 20 min at 37°C in a water bath with intermittent shaking. After the waiting time for the trypsin digestion is over we added to the tissue-trypsin suspension a mixture of prewarmed DMEM/Dnase I (Sigma DN-25, Dnase I final concentration 0. 25 mg/ml) followed by an incubation for 4 min at 37°C. The resulting suspension was dispersed by a mild mechanical trituration which consisted in the passage through 18-, 22- and 25- gauge needles. This cell suspension was then filtred through 70 μm strainer (BD Falcon 352350). After extensive washs in prewarmed HBSS, these dissociated cells were resuspended and plated in 75-cm^2 ^Falcon tissue-culture flasks (BD Biosciences) previously coated with 10 μg/ml poly-D-lysine (PDL). These mixed cells were growing at 37°C and 5% CO_2 _in DMEM (Gibco) supplemented with 10% FBS, penicillin (100 units/ml), and streptomycin (100 mg/ml). The media was changed every 2 or 3 days thereafter.

At 10 days-*in-vitro*, a confluent monolayer of astrocytes was apparent, on top of which oligodendrocyte precursor cells and a loosely attached layer of phase-bright microglia was obtained. Microglias were collected by shaking the flasks for 1 h at 200 rpm at 37°C and 5% CO_2_. Dislodged cells were resuspended and grown in culture medium for microglia [RPMI medium 1640 (Gibco) supplemented with 10% FBS, L-glutamine (1 mM), sodium pyruvate (1 mM), penicillin (100 units/ml), and streptomycin (100 mg/ml)]. The cells were allowed to adhere to the surface of PDL-coated coverslips (30 min at 37°C and 5% CO_2_), and nonadherent cells were rinsed off.

#### Microglia cells preparation for confocal microscopy

Briefly, confluent cells were exposed to 300 nM of BK for 24 h to induce B_1_R [50,51]. Control cells were exposed to vehicle. After incubation with BdABK, cell were washed, then fixed and permeabilized with 100% methanol previously stored at -20°C. The fixed cells were then processed as described for immunostaining.

#### Thermal hyperalgesia

Thermal hyperalgesia was assessed according to the method described by Hargreaves et al., 1988 [52] with minor modifications. Briefly, rats were placed (unrestrained) within a Plexiglass enclosure on a transparent glass floor and allowed to acclimatize for 20–30 min. An infrared beam that constitutes the noxious heat stimulus (Plantar test, Ugo Basile, Italy) was moved beneath the plantar surface of the hind paw. Thermal nociceptive threshold was defined as the latency (seconds) between the heat stimulus (46°C) onset and the paw withdrawal using a feedback-controlled shut-down unit. A cut-off time of 33 s was used to avoid tissue damage. Each paw was tested three times alternatively at minimum intervals of 3 min between stimulation to avoid sensitization of the hind paw. The rats were trained on several days prior to testing B_1_R agonists. Thereafter, the thermal nociceptive threshold was assessed on 3 consecutive days as follows: day 1: baseline, saline and the first dose of des-Arg^9^-BK and BdABK (22.5 μg/kg); day 2: des-Arg^9^-BK and BdABK (225 μg/kg); day 3: des-Arg^9^-BK and BdABK (2250 μg/kg). Agonists were injected intraperitoneally at 1 h apart. This series of experiments was conducted in 3 control and 3 STZ-diabetic rats because the quantity of BdABK available for *in vivo *study was restricted. Thermal hyperalgesia was calculated as a percentage of the maximum possible effect (% MPE) according to the following formula: % MPE = (100 × (drug latency minus baseline latency)/(cut-off time minus baseline latency)) [9]. The baseline latency corresponds to the average of the first three measurements.

### Statistical analyses

All data were expressed as means ± S.E.M. obtained from *n *rats. Statistical significance was determined with Student's *t*-test for unpaired samples or a one-way analysis of variance (ANOVA) followed by post-hoc Dunnett test for multiple comparisons. IC_50 _values were calculated by Graph Pad Prism 4.0 (GraphPad software, USA). Only probability (P) values less than 0.05 were considered to be statistically significant.

## Results

### B_1_R fluorescent labeling and selectivity of BdABK

**Figure 1:**
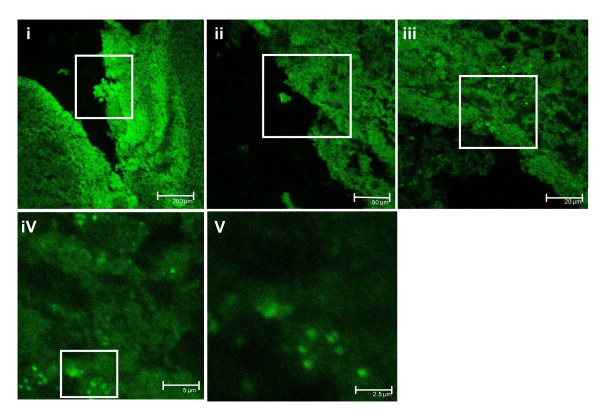
**B_1_R distribution in thoracic spinal cord of STZ-treated rats was shown by confocal microscopy with [Nα-Bodipy]-des-Arg^9^-BK**. Shown are pictures from low (i) to high magnification (V) of the dorsal horn. Scale bars = 200, 50, 20, 5 and 2.5 μm, respectively from (i) to (V). Pictures are representative of a minimum of 4 sections per rat from 4 different STZ-diabetic rats.

**Figure 2:**
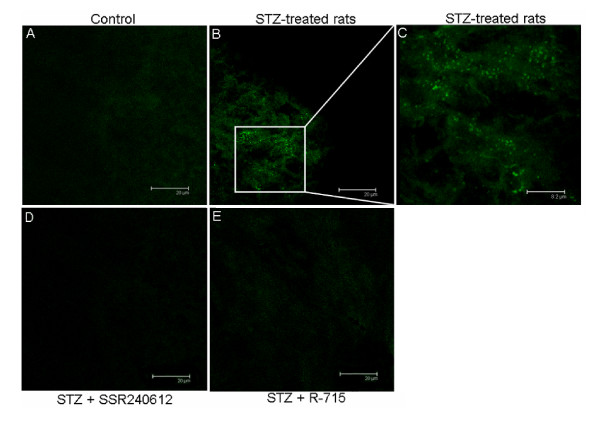
**[Nα-Bodipy]-des-Arg^9^-BK (BdABK) selectivity for B_1_R was evaluated by confocal microscopy**. While B_1_R labeling in the presence of BdABK was absent in thoracic spinal cord of control rats (A), it was shown as green dots in STZ-treated spinal cord (B, and enlarged in C). B_1_R labeling was absent in STZ-treated spinal cord when BdABK (50 μM) was co-incubated with 10^-5^M SSR240612 (D) or 10^-5^M R-715 (E). Background staining represents non specific autofluorescence. Scale bars = 20 μm (A, B, D, E) and 8.2 μm (C). Pictures are representative of a minimum of 4 sections per rat from 4 different STZ-diabetic rats.

Figure 1 illustrates B_1_R labeling with BdABK from low (i) to high (V) magnification (green dots) in dorsal horn of thoracic spinal cord of STZ-treated rats. As depicted in Figure 2, BdABK showed no labeling in control thoracic spinal cord (A), while the labeling of B_1_R was apparent in thoracic spinal cord of STZ-treated rats as revealed by green dots (B). Selectivity and specificity of the labeling were demonstrated by the absence of BdABK labeling in STZ-spinal cord sections when the B_1_R antagonists SSR240612 (D) and R-715 (E) were added at 10^-5^M.

### B_1_R and B_2_R binding and IC_50 _value of BdABK

**Figure 3:**
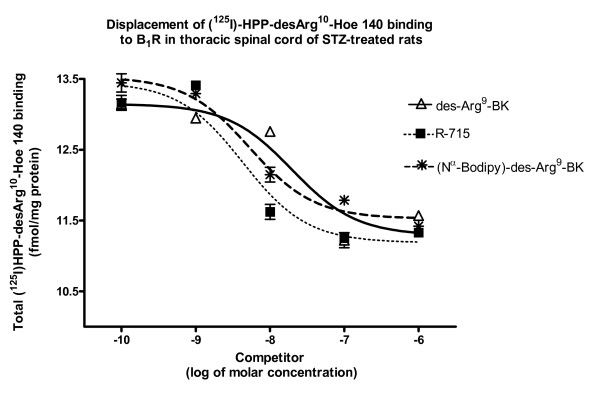
**[Nα-Bodipy]-des-Arg^9^-BK selectivity for B_1_R was evaluated by quantitative autoradiography**. R-715 (selective B_1_R antagonist), des-Arg^9^-BK (selective B_1_R agonist) and [Nα-Bodipy]-des-Arg^9^-BK (fluorescent agonist of B_1_R) displaced in a concentration-dependent manner, from 10^-10 ^to 10^-6 ^M, the total binding of 200 pM [^125^I]-HPP-desArg^10^-Hoe 140 to B_1_R. Data are means ± SEM of 4 sections per rat from 7 different rats for each compound.

**Figure 4:**
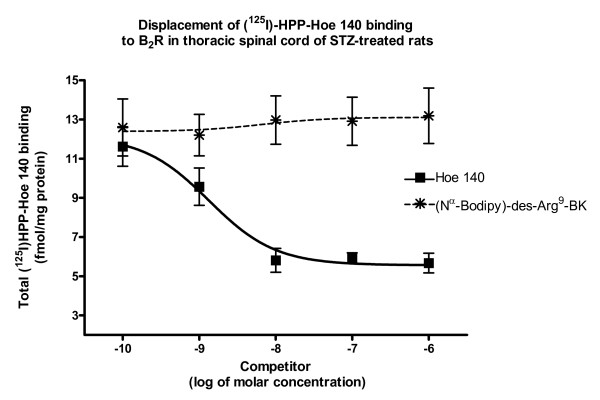
**[Nα-Bodipy]-des-Arg^9^-BK affinity for B_2_R was evaluated by quantitative autoradiography**. Increasing concentration (10^-10 ^to 10^-6 ^M) of Hoe 140 (selective B_2_R antagonist) displaced total binding of 200 pM [^125^I]-HPP-HOE-140 to B_2_R. In contrast, same concentrations of [Nα-Bodipy]-des-Arg^9^-BK (fluorescent B_1_R agonist) did not displace the B_2_R radioligand. Data are means ± SEM of 4 sections per rat from 7 different rats for each compound.

Competition experiments using 200 pM [^125^I]-HPP-desArg^10^-Hoe 140 and 10^-10 ^to 10^-6 ^M of des-Arg^9^-BK, R-715, or BdABK revealed that kinin analogues decreased in a concentration-dependent manner the binding of [^125^I]-HPP-desArg^10^-Hoe 140 in the thoracic spinal cord of STZ-treated rats (Fig. 3). The rank order of potency to inhibit total B_1_R binding sites was R-715 = BdABK > des-Arg^9^-BK with IC_50 _values of 4.3 ± 0.2 nM, 5.3 ± 0.1 nM and 19 ± 0.2 nM, respectively. In contrast, BdABK (10^-10 ^to 10^-6 ^M) failed to inhibit the binding of 200 pM [^125^I]-HPP-Hoe 140 to B_2_R in the thoracic spinal cord of STZ-treated rats (Fig. 4). In comparison, same concentrations of Hoe 140 displaced B_2_R binding sites with IC_50_value of 1.33 ± 0.1 nM.

### BdABK mediated *in vivo *thermal hyperalgesia

**Figure 5:**
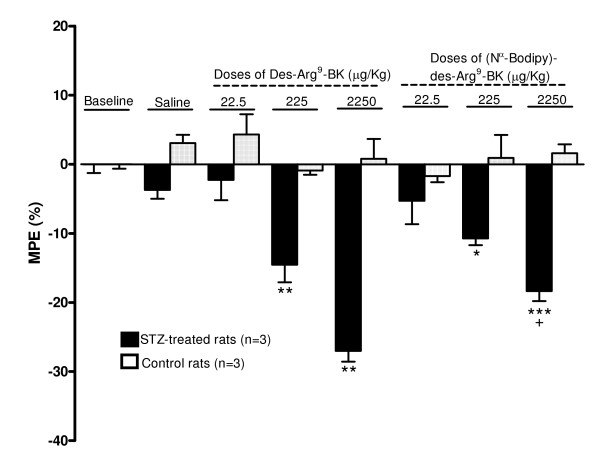
**The ability of intraperitoneally injected [Nα-Bodipy]-des-Arg^9^-BK and its native agonist, des-Arg^9^-BK, to alter the paw withdrawal threshold in STZ-treated and control rats**. Data represent maximal effects and are the average of 3 readings taken at 9, 12 and 15 min post-injection in 3 rats per group. Statistical comparison to control (*) and 2250 μg/kg des-Arg^9^-BK (+) are indicated by * + P < 0.05; ** P < 0.01; *** P < 0.001.

**Figure 6:**
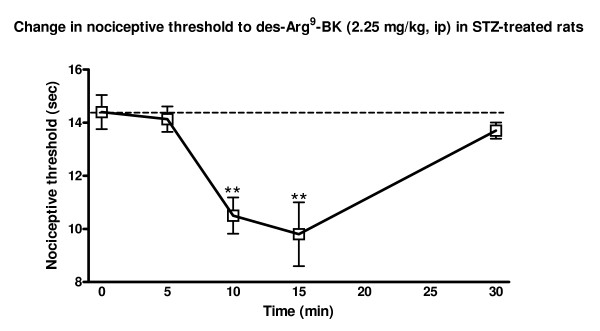
**Time-course effect of des-Arg^9^-BK (2.25 mg/kg, i.p.) on the nociceptive threshold in STZ-treated rats**. Data are means ± SEM of 3 rats. Statistical comparison to time 0 (*) is indicated by ** P < 0.01.

The *in vivo *effect of BdABK on pain behavior was assessed by determining its ability to induce thermal hyperalgesia upon intraperitoneal injection in STZ-treated rats. As expected, BdABK and des-Arg^9^-BK had no significant effect on the nociceptive threshold in control rats, yet both agonists caused thermal hyperalgesia in STZ-diabetic rats at 0.225 and 2.25 mg/kg. These effects were dose-dependent and significant when compared to saline or control (Fig. 5). BdABK was however slightly but significantly less potent than des-Arg^9^-BK to induce hyperalgesia at the highest dose. As exemplified by des-Arg^9^-BK, this response peaked at 15 min post-injection and was reversible after 30 min (Fig. 6).

### B_1_R mRNA expression assessed by qPCR

**Figure 7:**
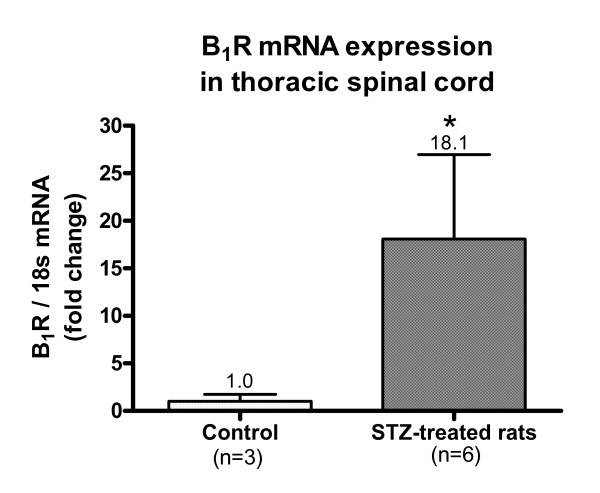
**B_1_R mRNA expression in STZ-treated and control thoracic spinal cords was measured by quantitative real-time PCR**. Data are means ± SEM of (3 to 6) rats. Statistical comparison with control is indicated by * P < 0.05.

A low basal expression of kinin B_1_R mRNA was detected in the spinal cord of control rats (Fig. 7). This expression was significantly increased (18-fold) in the spinal cord of STZ-diabetic rats.

### Density of B_1_R binding sites assessed by quantitative autoradiography

**Figure 8:**
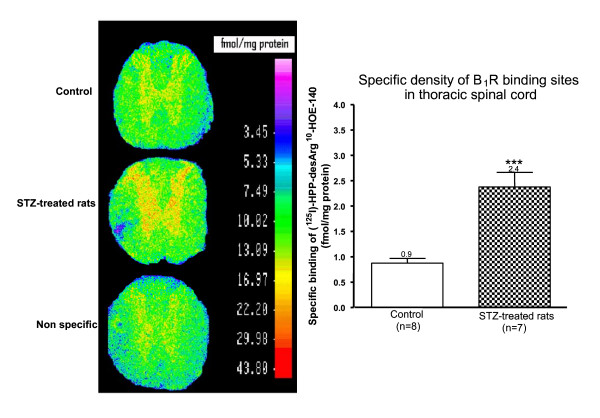
**B_1_R binding sites in STZ-treated and control thoracic spinal cords were measured by quantitative autoradiography**. Specific density of B_1_R binding sites are means ± SEM of (7 to 8) rats. Statistical comparison with control is indicated by *** P < 0.001.

As presented in Figure 8, quantitative *in vitro *autoradiography showed an increase density of specific B_1_R binding sites throughout the grey matter of the thoracic spinal cord in STZ-treated rats when compared to age-matched control spinal cord. B_1_R binding sites (2.4 fmol/mg protein) in spinal cord of STZ-treated rats were 2.7-fold greater than those measured in control rats (0.9 fmol/mg protein).

### B_1_R colocalized on microglial cells in thoracic spinal cord

**Figure 9:**
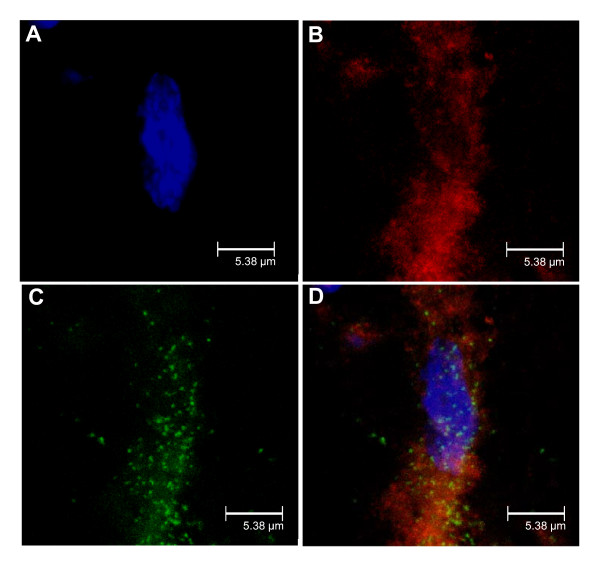
**STZ-treated thoracic spinal cord was exposed to TOPRO-3, a specific fluorescent dye for DNA (A)**. Microglia was identified with anti-IBA-1 (B). The B_1_R was stained with the selective fluorescent agonist, BdABK (C). Colocalization of the three markers is shown in panel D. TOPRO-3 dye is blue (ext: 642 nm/em: 661 nm), anti-IBA-1 dye is red (ext: 550 nm/em: 570 nm) and BdABK dye is green (ext: 505 nm/em: 515 nm). Scale bar = 5.38 μm. Pictures presented are representative of a minimum of 4 sections per rat from 4 different animals.

Figure 9 shows the colocalization of BdABK, TOPRO-3 and anti-IBA-1 in STZ thoracic spinal cord. Data suggest that B_1_R is present on spinal microglial cells in STZ-diabetic rats.

### B_1_R colocalized in primary cultured microglial cells

**Figure 10:**
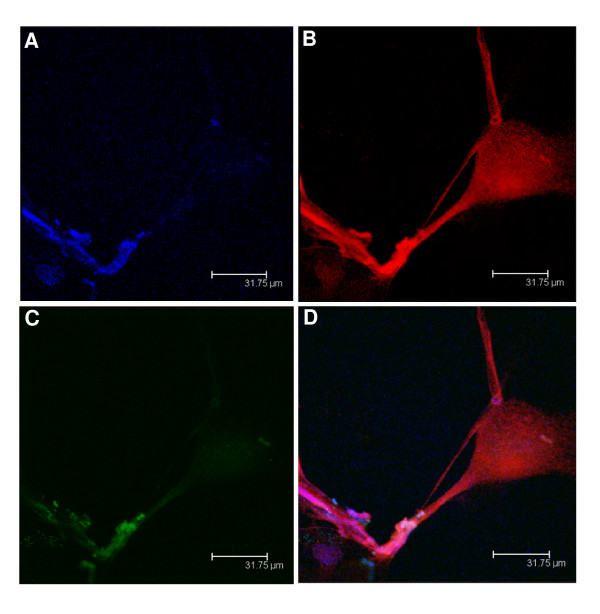
**Rat microglial primary cultured cells were exposed for 24 h to 300 nM BK to increase B_1_R expression**. Then, they were exposed to TOPRO-3, a specific fluorescent dye for DNA (A), to anti-IBA-1, a specific antibody against microglia (B) and BdABK to stain B_1_R (C). Colocalization of the three markers is shown in panel D. TOPRO-3 dye is blue (ext: 642 nm/em: 661 nm), anti-IBA-1 dye is red (ext: 550 nm/em: 570 nm) and BdABK dye is green (ext: 505 nm/em: 515 nm). Scale bar = 31.75 μm. Pictures presented are representative of 4 cultured cells samples from 4 different animals.

Figure 10 shows the colocalization of BdABK, TOPRO-3 and anti-IBA-1 in primary microglial cell culture. B_1_R was induced by a pre-treatment with 300 nM BK. About 95 ± 2% of the primary cell culture showed a positive labeling with anti-IBA-1 confirming cell purity. Data suggest that B_1_R can be induced *in vitro *on microglial cells.

### B_1_R colocalized on sensory C fibers in thoracic spinal cord

**Figure 11:**
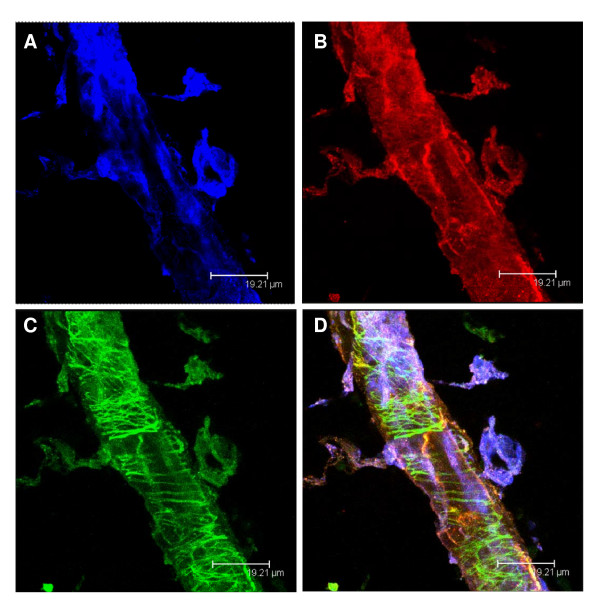
**STZ-treated spinal cord was exposed to anti-CGRP, a selective antibody of sensory C fibers (A)**. TRPV1 was labeled with anti-TRPV1 another marker of sensory C fibers (B). The B_1_R was stained with the selective fluorescent agonist, BdABK (C). Colocalization of the three markers is shown in panel D. Anti-CGRP dye is blue (ext: 650 nm/em: 680 nm), anti-TRPV1 dye is red (ext: 550 nm/em: 570 nm), and BdABK dye is green (ext: 505 nm/em: 515 nm). Scale bar = 19.21 μm. Pictures presented are representative of a minimum of 4 sections per rat from 4 different animals.

Figure 11 shows the colocalization of BdABK, anti-TRPV1 and anti-CGRP in the thoracic spinal cord of STZ-treated rats. Data suggest that B_1_R and TRPV1 are co- localized on sensory C fibers in STZ-diabetic rats.

### B_1_R colocalized on astrocytes in thoracic spinal cord

**Figure 12:**
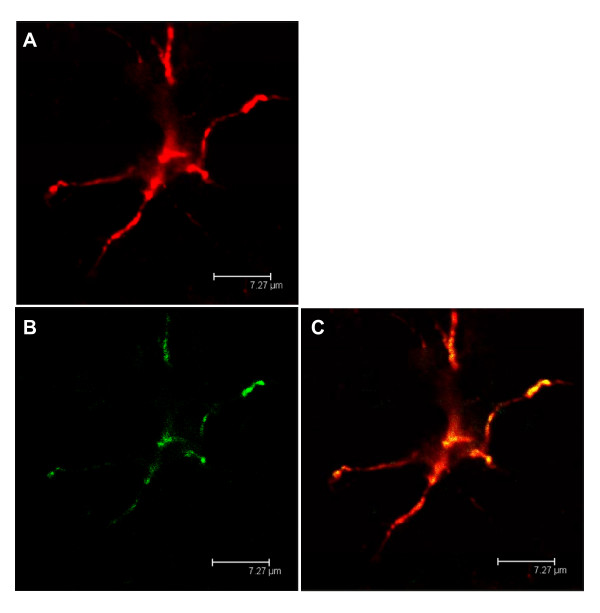
**STZ-treated spinal cord was exposed to anti-GFAP, a specific antibody against astrocytes (A) and to BdABK (B)**. Colocalization between B_1_R and the astrocytes is shown in panel C. Anti-GFAP dye is red (ext: 550 nm/em: 570 nm) and BdABK dye is green (ext: 505 nm/em: 515 nm). Scale bar = 7.27 μm. Pictures presented are representative of a minimum 4 sections per rat from 4 different animals.

Figure 12 shows the colocalization of BdABK and anti-GFAP in the spinal cord of STZ-treated rats. Data suggest that B_1_R is also present on spinal astrocyte cells in STZ-diabetic rats.

## Discussion

This study is using a newly developed selective and high affinity fluorescent ligand enabling the cellular localization of B_1_R on unfixed tissue. It provides the first evidence that B_1_R is localized on microglial cells, astrocytes and sensory C fibers in the thoracic spinal cord of STZ-diabetic rats. This study also highlights the early upregulation of B_1_R (mRNA and binding sites) in the thoracic spinal cord of hyperglycaemic STZ-treated rats.

### Diabetes induces B_1_R expression

STZ-diabetic rats provide an accessible model for studying the expression, the pharmacology and physiopathology of the B_1_R in the central nervous system. Pharmacological data showed that functional B_1_R was expressed in spinal cord of STZ-treated rats; its spinal activation led to sympathetically mediated increases of blood pressure and heart rate [53] and to thermal hyperalgesia [9]. Further autoradiographic and functional evidence for B_1_R induction was demonstrated in the lung [54], spinal cord [33], retina [6,55] and brain [56] of STZ-diabetic rats. However, this is the first report on mRNA expression in thoracic spinal cord of STZ-diabetic rats by qPCR. Hyperglycaemia associated with type 1 diabetes can activate NF-κB [57] which is known to induce B_1_R [2,3,58]. Moreover, oxidative stress associated with diabetes was reported to be involved in the induction of B_1_R [6,7,29,59].

### [N^α^-Bodipy]-des-Arg^9^-BK selectivity for B_1_R

Experiments by autoradiography confirm that BdABK is highly selective for B_1_R and does not bind to B_2_R. Indeed, BdABK failed to displace the B_2_R radioligand [^125^I]-HPP-Hoe-140 while it displaced the B_1_R radioligand, [^125^I]-HPP-desArg^10^-Hoe 140, with an IC_50 _of 5.3 ± 0.1 nM in thoracic spinal cord of STZ-treated rats. Results also evidenced that B_2_R binding sites were displaced by the selective antagonist, Hoe 140, with an IC_50 _value of 1.3 ± 0.1 nM while B_1_R binding sites were displaced by the natural B_1_R agonist, des-Arg^9^-BK (IC_50 _= 19 ± 0.2 nM) and by R-715, a selective B_1_R peptide antagonist (IC_50 _= 4.3 ± 0.2 nM). Comparison of IC_50 _values suggests that the affinity of the B_1_R agonist is increased by the addition of the Bodipy molecule. The stabilization of the N-terminus part of the peptide may contribute to prevent its degradation.

The reason for using 50 μM BdABK was based on preliminary study. The concentration of fluorescent probe needed to get a consistent labeling was higher than the IC_50 _value most likely because BdABK binds to B_1_R non-covalently and can be eliminated during the washout period of tissue sections. Signal amplification with radioactivity is also expected to be greater than that achieved with a fluorescent probe. BdABK showed no labeling in thoracic spinal cord of control rats which is in accordance with the inducible character of the B_1_R and its virtual absence in healthy tissues. The elimination of B_1_R labeling with BdABK after co-incubation with R-715 or SSR240612 confirms the specificity of the B_1_R fluorescent ligand.

Interestingly, BdABK maintained its biological activity as B_1_R agonist *in vivo*. Data obtained on the Hargreaves test revealed that BdABK was only slightly less potent than des-Arg^9^-BK to cause thermal hyperalgesia upon peripheral administration. This is consistent with the transient thermal hyperalgesia previously reported in the tail-flick test after intrathecal injection of des-Arg^9^-BK in rats made diabetics with STZ 24 h earlier [9]. Likewise, Gabra and Sirois [24] showed that intraperitoneal administration of des-Arg^9^-BK (400 μg/kg) in STZ-treated rats significantly reduced the paw withdraw threshold in the hot plate and tail-flick test.

### Localization of B_1_R

#### B_1_R on microglial cells

Previous work by Noda and coworkers [50,51] showed that B_1_R can be expressed in cultured rat microglia exposed to BK. We confirmed this result by using our fluorescent ligand in the same condition, thus providing additional evidence of its ability to bind B_1_R in a pure rat microglia model. BK acting via B_2 _receptors induces elevation of intracellular calcium leading to the phosphorylation and activation of NF-κB by protein kinase C [60]. NF-κB upregulates B_1_R upon binding to its nuclear promoter [2].

A recent study has demonstrated that B_1_R is involved in microglial migration toward rat brain lesion sites [61]. The presence of B_1_R on spinal microglial cells is in keeping with a recent study suggesting that activated dorsal horn microglia is a crucial component of STZ-induced tactile allodynia, mediated in part, by extracellular signal-regulated protein kinase signaling [62]. Importantly, the development of tactile and cold allodynia in a rat model of insulin-resistance was blocked by the B_1_R antagonist SSR240612 [28] and by two antioxidants (N-acetyl-L-cysteine and alpha-lipoic acid) known to prevent the induction of B_1_R [7,29]. Taken together, these results suggest a critical role for microglial B_1_R in generation of tactile allodynia, a manifestation of pain polyneuropathy. It is possible that microglial B_1_R is also involved in STZ-induced thermal hyperalgesia as this response was abolished by B_1_R antagonists [5,24,27] and was absent in B_1_R knockout mice treated with STZ [26].

#### B_1_R on astrocytes

In addition, the present study provides the first evidence that thoracic spinal cord astrocytes bear the B_1_R in STZ-diabetic rats. Astrocyte B_1_R may represent another target for neuropathic or chronic pain. Emerging evidence suggests a critical role for astrocytes in the passage from acute to chronic and neuropathic pain. It seems that intracellular calcium level oscillation in astrocytes could spread through astrocytal network and thereby facilitate the formation of new synapses. These new synapses could establish neuronal contacts for maintaining and spreading pain sensation [63]. Moreover, astrocytes are known to release various inflammatory mediators that promote neuroimmune activation and can sensitize primary afferent sensory neurons contributing to development of neuropathic pain [64].

#### B_1_R on sensory C fibers

Immunohistochemical data showed the presence of B_1_R in DRG and superficial laminae of spinal cord dorsal horn [30-32]. Those studies suggested a basal expression of B_1_R in primary sensory C fibers of normal rat. This is consistent with the expression of B_1_R in sensory C fibers of STZ-treated rats as revealed by the co-localization of B_1_R, CGRP and TRPV1. Horowitz [65] described the crucial role of small A-delta and C fibers in generation of diabetic polyneuropathy and their sensitivity to hyperglycaemia. Ueda's studies [66,67] support the hypothesis that the generation of neuropathic pain is related to alterations in gene and protein expression in primary sensory neurons which could contribute to demyelination of A-delta fibers through the down-regulation of myelin protein such as MBP, MPZ and PMP22. Demyelinated A-delta fibers sprout and synapse with A-beta fibers resulting in the enhancement of pro-nociceptive neurotransmitter release which generated allodynia. The presence of B_1_R on sensory C fibers is in agreement with an earlier pharmacological study that showed that the stimulation of B_1_R with an agonist in the spinal cord of STZ-diabetic rats provokes thermal hyperalgesia via the release of substance P [9].

#### Basal B_1_R expression in control rats

Authors failed to observe specific fluorescent labelling for B_1_R in normal rats which is rather consistent with the negligible level of B_1_R mRNA and binding sites. Moreover, intrathecal injection of B_1_R agonists or antagonists failed to cause behavioural, cardiovascular or nociceptive responses in control rats, suggesting that the basal expression of B_1_R is not functional in naïve rats [9,53]. Thus the function of the B_1_R detected by immunohistochemistry in the spinal cord of rodents and human remains elusive. It is feasible that B_1_R in control animals is uncoupled to G protein as demonstrated for other G-protein-coupled receptors [68,69]. Although it is possible that the immunological approach is more sensitive, we have evidence (unpublished data) showing that the commercially available B_1_R antibodies (M-19) from SantaCruz Biotechnologies (Santa Cruz, CA, USA) are not specific for immunohistochemical detection since B_1_R labeling persists in spinal cord isolated from B_1_R knockout mice. The latter B_1_R antibodies remain however suitable for Western blot analysis, suggesting that immunohistochemical studies reported with B_1_R antibodies remain to be validated with the appropriate controls in mutant mice.

## Conclusion

[Nα-Bodipy]-des-Arg^9^-BK was found selective for B_1_R with an IC_50 _value of 5.3 ± 0.1 nM in the rat spinal cord. Furthermore, BdABK maintains its biological activity as agonist as evidenced by its ability to induce thermal hyperalgesia in STZ-treated rats. This new fluorescent ligand enabled the detection of B_1_R in primary microglial cell culture and on microglial cells, astrocytes and sensory C fibers in the thoracic spinal cord of STZ-diabetic rats. Because all these cells have been implicated in neuropathic pain, the induction and up-regulation of the B_1_R on these elements consolidate the idea that kinin B_1_R is an important target for drug development in pain processes.

## List of abbreviations

B_1_R: kinin B_1 _receptor; STZ: streptozotocin; qPCR: quantitative real-time PCR; BK: Bradykinin; BdABK: [Nα-Bodipy]-des-Arg^9^-BK; BSA: bovine serum albumin; anti-IBA-1: anti-Ionized calcium binding adapter molecule 1; anti-GFAP: anti-Glial fibrillary acidic protein; anti-CGRP: anti-calcitonin-gene-related peptide; anti-TRPV1: anti-transient receptor potential vanilloid 1.

## Competing interests

The authors declare that they have no competing interests.

## Authors' contributions

ST performed animal treatments, Hargreaves test, real-time PCR analysis, confocal microscopy experiments and draft the manuscript. PTT helped designed the confocal microscopy protocol. DL performed *in vitro *microglia experiments. JS made cryostat tissue sections and autoradiography experiments. PG synthesized the fluorescent agonist. RC designed the study and revised the manuscript.
